# Spiritual and religious beliefs: do they matter in the medication adherence behaviour of hypertensive patients?

**DOI:** 10.1186/1751-0759-7-15

**Published:** 2013-10-18

**Authors:** Irene Kretchy, Frances Owusu-Daaku, Samuel Danquah

**Affiliations:** 1 Department of Clinical and Social Pharmacy, Faculty of Pharmacy and Pharmaceutical Sciences, Kwame Nkrumah University of Science and Technology, Kumasi, Ghana; 2 Department of Pharmacy Practice and Clinical Pharmacy, University of Ghana School of Pharmacy, College of Health Sciences, Accra, Ghana; 3 Department of Psychology, University of Ghana, Legon, Ghana

**Keywords:** Spirituality, Religiosity, Medication non-adherence, Co-morbidity, Ghana

## Abstract

**Background:**

Medication non-adherence is often a predominant problem in the management of hypertension and other chronic conditions. In explaining health behaviours, social determinants like spirituality and religiosity are increasingly identified to impact health and treatment. Although a number of researchers have found spirituality and religiosity to be primary resources among persons dealing with chronic disability and illness, studies relating this specifically to medication adherence have been limited.

**Methods:**

Our study sought to examine the interrelationship between spirituality/ religiosity and medication adherence among 400 hypertensive patients 18 years old and above. Spiritual Perspective Scale, Duke Religion Index, and the Morisky Medication Adherence Scale were used to determine spirituality, religiosity and medication adherence respectively.

**Results:**

The majority (93.25%) of patients poorly adhered to their medications. While high spiritual and religious beliefs formed core components of the lifestyles of patients, spirituality (*p* = 0.018) and not religiosity (*p* = 0.474) related directly with medication non-adherence. Likewise, after controlling for demography and other medical co-morbidities, patients with high spirituality were 2.68 times more likely to be poorly adherent than patients who place lower emphasis on the association between spirituality and health.

**Conclusion:**

Our study suggests that while spirituality/ religiosity was dominant among hypertensive patients, these spiritual attachments of patients with a supreme being potentially increased their trust in the expectation of divine healing instead of adhering adequately with their anti-hypertensive medications.

## Background

Medication adherence is a major health behaviour noted to improve the quality of life of patients. However, non-adherence is often a predominant problem in the management of hypertension and other chronic conditions [1]. Even though the World Health Organization has estimated the non-adherence rate to pharmacotherapy for hypertension to be approximately 50%, Ghana, a developing country has recorded a very high non-adherence rate of 93% [2].

Contrary to the availability of treatment for hypertension, most patients do not follow recommendations by their healthcare professionals and this results in poor hypertension management [3]. Most patients require one or more antihypertensive medications to achieve optimal blood pressure and this has been associated with an increased cost of hypertension management in Ghana [2]. A significant worsening of disease, treatment failures, increased hospitalizations, death, and increased health care costs remain the main consequences of medication non-adherence [4]. Patients with untreated and uncontrolled hypertension are at an increased risk of developing stroke, heart attack, and kidney or heart failure, which can eventually lead to death. Inadequate control of hypertension has also been associated with cognitive decline in elderly patients [5] resulting in both social and financial burdens [6].

In explaining health behaviours, social determinants like spirituality and religiosity are increasingly identified as impacting health and treatment. Systematically, spirituality and religiosity have been introduced into the medical field implying a growing interest in the possible perceived health benefits connected with having a spiritual belief and following a religious lifestyle [7].

Specifically, spirituality relates to feelings or experiences of reverence, peace, or attachment with a Supreme Being and is usually identified as that which gives a transcendent meaning to life where God takes the initiative in this typical divine-human relationship [8]. Notwithstanding varied descriptions of spirituality, the core concepts are the same [9]. It may well comprise the internal, personal, and emotional expression of the revered and is evaluated using spiritual wellbeing, peace, and comfort derived from faith, and spiritual coping [8].

Religiosity relates more with cultural and social norms and refers to an outward prescribed system of beliefs and guidelines of conduct [10]. It is generally explained in a behavioural context where rituals and other related symbolic activities (e.g., meditations, prayers, fasting, reading religious scripts, attendance at services, etc.) are practised by individuals according to their specific beliefs and modes of social organization [9,11]. These activities have been noted to strengthen the faith of people and assist them with decision making in health-related practices.

Formerly, spirituality and religiosity were examined as a one-dimensional construct, yet, various researchers have attempted to categorize and distinguish particular aspects of the two constructs [12]. Nevertheless, religion is generally inter-related with spirituality since the former provides a structured environment for spiritual exploration and practices in life and the two constructs have been conceptualized to influence the development of each other [13]. For example, religious practices encourage spiritual growth and spiritual activities are often an important aspect of religious participation [9,14].

Although there are still deliberations on possible association of spirituality/ religiosity with health and weaknesses in the definition and measurement of the constructs, attempts have been made to operationalize and conceptualize spirituality and religiosity to relate with health outcomes as well as its impact on the social domain [9,15-18].

Various studies have explored the influence of spirituality/religiosity on positive health behaviours among college students, in the field of gerontology, in heart failure, mental health outcomes, blood pressures, cancer and mortality rates [19-25] with other researchers not observing this trend [26,27].

The 2010 national population census indicated that over 90% of Ghanaians identified with a form of religion, yet, there is paucity of information regarding the spiritual/ religious inclination and the health seeking behaviours of Ghanaians. Additionally, a number of researchers have found spirituality and religiosity to be a primary resource among persons dealing with chronic disability and illness however, studies relating this specifically to medication adherence have been limited. Nevertheless, addressing spiritual and religious needs of patients could enhance their medication adherence behaviour and thus, improve their health outcome [28].

In view of this, the study postulated that: *Explored dimensions of spirituality/religiosity will significantly influence the adherence behaviour of hypertensive patients with the expectation that high scores of spiritual beliefs and religious practices will positively influence the decision by patients to adhere to their antihypertensive medications.*

## Methods

### Study setting

This is a two-centre study, involving the two major tertiary hospitals in Ghana - Korle-Bu Teaching Hospital (KBTH), Accra and Komfo Anokye Teaching Hospital (KATH), Kumasi. The KBTH, the premier and largest teaching hospital, is located in the Accra District of Greater Accra Region. Being the only tertiary hospital in the southern part of Ghana, KBTH serves the people of Accra and surrounding urban towns located southwards. Komfo Anokye Teaching Hospital, the second largest hospital in the country, is another tertiary health institution located in Kumasi, the Regional Capital of Ashanti Region. It serves as the main referral facility for the northern (non-coastal) parts of Ghana. In selecting these two hospitals, the rationale was to recruit research participants with diverse spiritual/ religious behaviours who would fairly represent both southern and northern parts of Ghana to allow for generalization of results.

### Study participants

After obtaining informed consent from 200 recruited study participants from each study site, a quantitative approach was employed in this hospital-based cross-sectional study between May and October, 2012. Hypertensive patients were identified through the patient records folders and this information was substantiated by the patients themselves. Researcher-administered structured questionnaires were used to obtain information on demographic characteristics, spirituality, religiosity, and medication adherence. The participants included in this study were male and female Ghanaian patients who were 18 years and above, diagnosed with hypertension only or hypertension with other health conditions. Patients should have reported being prescribed at least one anti-hypertensive medication not less than six months prior to the time of data collection. Hypertensive patients who were not on any form of prescribed anti-hypertensive medications, as well as patients who were unable to respond to or understand the English language, in patients, and the incapacitated were excluded from this study.

### Measures

Spirituality, religiosity, and medication adherence were the variables measured in this study. The ten-item Spiritual Perspective Scale (SPS) is designed to measure perceptions of the extent to which participants hold certain spiritual views and engage in spiritually-related interactions. Each of the 10 items uses a 6-point Likert-type scale ranging from strongly disagree to strongly agree is scored using the mean. Scores above the mean indicate high spiritual involvement and those below the mean value indicate the reverse. The SPS has been used successfully in a wide variety of adult populations. Using the Cronbach’s alpha, reliability has consistently rated above 0.90 and average inter-item correlations range from 0.54 to 0.60 across adult groups [29].

Religious beliefs and religious involvement was measured with the Duke Religion Index (DUREL), a two-item measure assessing two domains of religiosity: Organized Religious Activity (ORA), i.e., (“How often do you attend church or religious meetings?”), and Non-organized Religious Activity (NORA), i.e., (“How often do you spend time in private religious activities, such as prayer, meditation, or Bible study?”). Responses range from 1 (“more than once a week”) to 6 (“never”) for ORA and 1 (“more than once a day”) to 6 (“rarely or never”) for NORA. Scoring is based on a separate regression model for each item. DUREL has been validated in health research with Cronbach’s alpha values ranging from 0.75 to 0.88 [30].

Medication adherence was assessed with the Morisky Medication Adherence Scale (MMAS). It has 8 items of that respondents score from zero to eight and enables categorization as low adherence (< 6), medium adherence (6 - < 8), and high adherence (8). Patients who scored low and moderate were grouped as poorly adherent to allow for statistical analysis [31]. The MMAS reliability measure was 0.83 for a study on hypertensive outpatients [32].

### Statistical analysis

Data for this study were analyzed using the Statistical Package for Social Sciences (SPSS) version 20. Descriptive statistics were used for frequency counts and percentages of characteristics of participants and study tools (SPS, DUREL and MMAS). Chi-square test and logistic regression were used for evaluating relationship spirituality/ religiosity and medication adherence.

### Ethical considerations

All procedures for the study were endorsed by the Institutional Review Boards at Noguchi Memorial Institute for Medical Research, Accra and Committee of Human Research, Publications and Ethics, Kumasi with the respective ethical approval codes NMIMR-IRB CPN 044/10-11 and CHRPE/AP/022/12. Written permission was sought from Prof. Pamela Reed and Dr. Donald Morisky to enable the research team use the SPS and MASS, respectively.

## Results

### Sample and subscale characteristics

A summary of the socio-demographic characteristics (mean and standard deviation) of the study population in relation to sex, age, and co-morbidities are presented in Table 1. The majority of patients were female (62.75%), married (63.50%), and between the ages of 50 and 59 years (32.50%) who had attained a minimum of secondary school education (54.25%), were Christians (90.0%) and had been diagnosed with hypertension for less than or equal to 10 years (79.50%). Forty two percent (42%) of the patients had co-morbidities and diabetes accounted for 60% of these. The various scales used in this study (SPS, DUREL and MMAS) showed good reliability coefficients (Table 2).

**Table 1:** Sample characteristics

**Variable**	**Mean**	**Standard deviation**
Sex	1.63	0.48
Age	57.10	10.87
Co-morbidities	1.58	0.49

**Table 2:** Characteristics of sub scales

**Scale**	**Number of items**	**Reliability coefficient**
SPS	10	0.878
DUREL	2	0.513
MMAS	8	0.793

### Medication adherence

Medication adherence behaviour of patients was assessed using MMAS. Table 3 shows that ninety-three percent of participants poorly adhered to their anti-hypertensive medications.

**Table 3:** Frequency distribution for medication adherence

**Question**		**Yes**	**No**
		**Freq. N (%)**	**Freq. N (%)**
1.	Do you sometimes forget to take your hypertension medications?	282 (70.5)	118 (29.5)
2.	Over the past two weeks, were there any days when you did not take your hypertension medicine?	328 (82.0)	72 (18.0)
3.	Have you ever cut back or stopped taking your medication without telling your doctor because you felt worse when you took it?	341 (85.25)	59 (14.75)
4.	When you travel or leave home, do you sometimes forget to bring along your medications?	327 (81.75)	73 (18.25)
5.	Did you take your high blood pressure medicine yesterday?	335 (83.75)	65 (16.25)
6.	When you feel like your blood pressure is under control, do you sometimes stop taking your medicine?	340 (85.0)	60 (15)
7.	Do you ever feel hassled about sticking to your blood pressure treatment plan?	337 (84.25)	63 (15.75)
8.	How often do you have difficulty remembering to take all your medications?	Freq.	Percentage
	oNever/Rarely	6	1.50
	oOnce in a while	6	1.50
	oSometimes	34	8.50
	oUsually	101	25.25
	oAll the time		
	Level of adherence		
	Low	323	80.75
	Moderate	50	12.50
	High	27	6.75
	Modified adherence categorization		
	Poor	373	93.25
	High	27	6.75

### Spirituality and religiosity

Very high proportions of patients frequently engaged in spiritually related activities, which formed significant aspects of their daily lives (Table 4). Similarly, Table 5 indicates that over 80% of the patients regularly (once or more than once a week) attended church or other religious meetings. The majority (> 50%) of patients frequently (daily or more than once a day) engaged in non-organized religious activities like prayer, meditation, or Bible study (Table 6).

**Table 4:** Frequency distribution for SPS

**Statement**	**Not at all**	**About once a year**	**About once a month**	**About once a week**	**About once a day**
1. In talking with family and friends, how often do you mention spiritual matters	32	10	39	121	198
2. How often do you share with others the problems and joys of living according to your spiritual beliefs	12	13	31	144	200
3. How often do you read spiritually-related material	45	14	11	89	241
4. How often do you engage in private prayer and meditation	6	8	14	57	315
5. Forgiveness is an important part of my spirituality	6	7	12	52	323
6. I seek spiritual guidance in making decisions in my everyday life	7	7	21	57	308
7. My spirituality is a significant part of my life	8	6	8	59	319
8. I frequently feel very close to God or ‘a higher power’ in prayer, during public worship or at important moments in my life	8	12	23	43	314
9. My spiritual views have had an influence upon my life	12	4	10	84	290
10. My spirituality is especially important to me because it answers many questions about the meaning of life	6	4	10	53	327

**Table 5:** Distribution of organised religious activity by patients

**Item**	**More than once/week**	**Once a week**	**A few times a month**	**A few times a year**	**Once a year or less**	**Never**
How often do you attend church or other religious meetings?	178	176	17	10	8	11

**Table 6:** Distribution of non-organised religious activity by patients

**Item**	**More than once/day**	**Daily**	**Two/more times/week**	**Once a week**	**A few times a month**	**Rarely or Never**
How often do you spend time in private religious activities, such as prayer, meditation or Bible study?	135	113	66	59	10	17

### Spirituality/ religiosity and medication non-adherence

The relationship between spirituality, religiosity, and medication non-adherence was measured with a chi-square test and logistic regression. After adjusting for demographic characteristics and co-morbid health conditions, spirituality, but not religiosity, was associated with medication non-adherence, although patients exhibited high levels of both spirituality and religiosity at p < 0.05 (Tables 7 and 8). Additionally, a logistic regression analysis conducted to determine whether the presence of co-morbidities considerably influenced the medication adherence of respondents revealed no significant association [(OR) = 1.31 (95% CI 0.60 – 2.86), *p* = 0.504].

**Table 7:** Relationship between spirituality/religiosity and medication non-adherence

**Medication adherence**	**Spirituality (%)**		**ORA (%)**		**NORA (%)**	
	**Low**	**High**	**Low**	**High**	**Low**	**High**
High	51.85	48.15	33.33	66.67	29.63	70.37
Poor	30.03	60.07	45.31	54.69	34.05	65.95
X^2^	5.558		1.494		0.222	
*p* value	0.018		0.474		0.895	

*p* < 0.05.

**Table 8:** Logistic regression model for spirituality, organised and non-organised religiosity in relation to medication non-adherence behaviour

**Variable**	**Odds ratio**	**95% confidence interval**	***p*value**
High spirituality (Ref: Low spirituality)	2.68	1.20 –5.96	0.016
High ORA (Ref: Low ORA)	0.65	0.35 – 1.21	0.176
High NORA (Ref: Low NORA)	1.04	0.63 – 1.71	0.874

## Discussion

The study found that poor adherence to anti-hypertensive medication regimen was high, occurring in 93.25% of respondents. This corroborates findings from a previous study by Buabeng et al. (2004) [2] that observed non-adherence rates by hypertensive patients to be essentially the same (93%), but is double the worldwide estimate of 50% put forward by the WHO. Although associations were not measured, it is possible to project that such a great difference in non-adherence could negatively impact health outcomes and contribute considerably to worsening of disease, increased health care costs, and eventually death [4].

The study has also demonstrated that a major proportion of hypertensive patients exhibited a high sense of spiritual belief and followed consistent religious lifestyles. Of the 400 hypertensive patients, about 90% were Christians, 5% were Muslims, while 1% identified with the Traditional religion. This trend is comparable to findings from the national population census in 2010, in which approximately 71%, 18% and 5% of the Ghanaian population were Christians, Muslims, and Traditionalists respectively. Similarly, a great proportion of patients regularly (once or more than once a week) attended church or other religious meetings, and a major percentage of the patients frequently (daily or more than once a day) engaged in non-organized religious activities like prayer, meditation, or Bible study. It was also apparent from the Spiritual Perspective Scale that very high proportions of patients frequently engaged in spiritually related activities. These spiritual activities formed noteworthy aspects of their daily lives. With regard to establishing a relationship between spirituality/ religiosity and anti-hypertensive medication adherence, we observed that while spirituality significantly influenced non-adherence, organized and non-organized religiosity did not. Likewise, after controlling for demography and co-morbidities, patients with high spirituality were 2.68 times more likely to be poorly adherent than patients who place lower emphasis on the association between spirituality and health.

This means that the lower likelihood of adhering to treatment was a result of high spiritual beliefs of patients and not behavioural religious characteristics. These outcomes directly contradict our postulation that ‘*spirituality/religiosity will significantly influence medication adherence positively’.* The results also contradict findings by Simoni, Frick, and Huang (2006) [33] and Raghavan et al. (2013) [34] whose studies have shown a positive impact of spirituality/ religiosity on medication adherence. It is worth noting that these studies were not conducted with a sub-Saharan African population. Spirituality/religiosity is an important component of the cultural beliefs of Africans, but to date there is little information examining how sub-Saharan Africans, and specifically Ghanaian adults with hypertension, relate spirituality/religiosity to medication adherence. For example, in examining barriers to antiretroviral therapy (ART) adherence in Uganda, Wanyama et al. (2007) [35] noted that some patients discontinued ART because of a belief in spiritual healing. In our study, we have demonstrated that although hypertensive patients exhibited high spiritual beliefs and engaged in frequent religious activities, spirituality and not religiosity influenced medication non-adherence. Thus, beliefs and practices can be treated as separate domains. These results seem to suggest that hypertensive patients related more to experiences of peace, and reverence or attachment with a Supreme Being [36]. Potentially, they may place more trust in the divine expectation of healing and not on conventional orthodox medications. The patients may tend to believe in God or a Supreme Being for possible healing, knowing the chronic and in most cases incurable nature of hypertension [35]. This further suggests that some patients would risk not taking their medications while anticipating divine healing outcomes. The observation is in line with spiritual causal theories strongly underlining chronic conditions in Ghana [37]. In a related study on medication adherence among persons with mental illness in Ghana, a similar trend was observed. Patients and their families sought spiritual interventions for their mental illness because they perceived the conventional antipsychotic medications failed to achieve a complete cure [38].

It is thus imperative for health professionals to be holistic in their approach to healthcare by taking into consideration the importance of the spirituality of their patients while providing care.

In spite of the above findings, the study was limited in the fact that the main target participants were derived from tertiary hospitals only, yet, hypertensive patients in Ghana are managed in various hospitals, not only the two tertiary hospitals used for this study.

## Conclusion

This study anticipated filling the knowledge gap in relation to establishing an association between spirituality/ religiosity and medication adherence among hypertensive patients in Ghana and by extension sub-Saharan Africans. The study outcome has demonstrated that while high spiritual and religious beliefs form core components of the lifestyles of hypertensive patients, spirituality, not religiosity, related directly to medication non-adherence, a highly prevalent risk behaviour of the patients. Thus, religiosity ought not to be equated to spirituality. Our study suggests that the reverence, experiences, and attachment of patients with a supreme being may potentially increase their trust or expectation of divine healing to the detriment of reliance on conventional orthodox anti-hypertensive medications. Thus, the study suggests that when treating patients or implementing medication non-adherence intervention programmes, considerable emphasis should be on the dynamics of the effects of spirituality, not necessarily religiosity.

## Competing interests

The authors declare that they have no competing interests.

## Authors’ contributions

IK was involved with research concept, data collection, data analysis, interpretation of results, and writing of manuscript. FO contributed to research concept, study design and review of manuscript. SD participated in research concept and study design. All authors reviewed and approved the final manuscript.

## References

[B1] Ho MP Bryson CL Rumsfeld JS Medication adherence: its importance in cardiovascular outcomes Circulation 2009 119 3028 3035 19528344 10.1161/CIRCULATIONAHA.108.768986

[B2] Buabeng KO Matowe L Plange-Rhule J Unaffordable drug prices: the major cause of non-compliance with hypertension medication in Ghana J Pharm Pharmaceut Sci 2004 7 3 350 352 15576016

[B3] Agyemang C Bruijnzeels MA Owusu-Dabo E Factors associated with hypertension awareness, treatment, and control in Ghana, west Africa J Hum Hypertens 2006 20 67 71 16121199 10.1038/sj.jhh.1001923

[B4] Osterberg L Blaschke T Adherence to medication N Engl J Med 2005 353 487 497 16079372 10.1056/NEJMra050100

[B5] Gombojav B Yi SW Sull JW Nam CM Ohrr H Combined effects of cognitive impairment and hypertension on total mortality in elderly people: the kangwha cohort study Gerontology 2011 57 6 490 496 21358170 10.1159/000323759

[B6] Redwood H Hypertension, society, and public policy Eur Heart J 2007 9 B13 B18

[B7] Penman J Oliver M Harrington A Spirituality and spiritual engagement as perceived by palliative care clients and caregivers Aust J Adv Nurs 2009 26 4 29 35

[B8] Kudel I Cotton S Szaflarski M Holmes WC Tsevat J Spirituality and religiosity in patients with HIV: a test and expansion of a model Ann Behav Med 2011 41 1 92 103 21103963 10.1007/s12160-010-9229-x

[B9] Miller WR Thoresen CE Spirituality, religion, and health Am Psychol 2003 58 24 35 12674816 10.1037/0003-066x.58.1.24

[B10] Mohr WK Spiritual issues in psychiatric care Perspect Psychiatr Care 2006 42 174 183 16916420 10.1111/j.1744-6163.2006.00076.x

[B11] Koenig HG McCullough ME Larson DB Handbook of religion and health 2001 Oxford: Oxford University Press

[B12] Pearce MJ Little TD Perez JE Religiousness and depressive symptoms among adolescents J Clin Child Adolesc Psychol 2003 32 2 267 276 12679285 10.1207/S15374424JCCP3202_12

[B13] McDade R Nielsen SM, Plakhotnik MS A mixed method study of spirituality and the influence on TB medication adherence Proceedings of the sixth annual college of education research conference: 2007: urban and international education section Miami, Florida: International University 54 59

[B14] Armstrong TD Crowther MR Spirituality among older African-Americans J Adult Dev 2002 9 1 3 12

[B15] Campbell JD Yoon DP Johnstone B Determining relationships between physical health and spiritual experience, religious practices, and congregational support in a heterogeneous medical sample J Relig Health 2010 49 3 17 20162451 10.1007/s10943-008-9227-5

[B16] Hackney CL Sanders GS Religiosity and mental health: a meta-analysis of recent studies J Sci Study Relig 2003 42 43 55

[B17] Hall DE Meador KG Koenig HG Measuring religiousness in health research: review and critique J Relig Health 2008 47 2 134 163 19105008 10.1007/s10943-008-9165-2 PMC8823950

[B18] Levin J “And let us make us a name”: reflections on the future of the religion and health field J Relig Health 2009 48 125 145 19291406 10.1007/s10943-009-9243-0

[B19] Buck AC Williams DR Musick MA Sternthal MJ An examination of the relationship between multiple dimensions of religiosity, blood pressure, and hypertension Soc Sci Med 2009 68 2 314 322 19019516 10.1016/j.socscimed.2008.10.010 PMC2654362

[B20] Gillum RF Ingram DD Frequency of attendance at religious services, hypertension, and blood pressure: the third national health and nutrition examination survey Psychosom Med 2006 68 3 382 385 16738068 10.1097/01.psy.0000221253.90559.dd

[B21] Hummer RA Ellison CG Rogers RG Moulton BE Romero RR Religious involvement and adult mortality in the United States: review and perspective South Med J 2004 97 1223 1230 15646761 10.1097/01.SMJ.0000146547.03382.94

[B22] Koenig HG Religion, spirituality, and medicine: research findings and implications for clinical practice South Med J 2004 97 1194 1200 15646757 10.1097/01.SMJ.0000146489.21837.CE

[B23] Clay KS Talley C Young KB Exploring spiritual well-being among survivors of colorectal and lung cancer J Relig Spiritual Soc Work 2010 29 1 14 32 20625520 10.1080/15426430903479247 PMC2900809

[B24] Powell LH Shahabi L Thoresen CE Religion and spirituality. Linkages to physical health Am Psychol 2003 58 1 36 52 12674817 10.1037/0003-066x.58.1.36

[B25] Riegel B Moser DK Anker SD Appel LJ Dunbar SB State of the science: promoting self-care in persons with heart failure: a scientific statement from the American heart association Circulation 2009 120 12 1141 1163 19720935 10.1161/CIRCULATIONAHA.109.192628

[B26] Westlake C Dracup K Creaser J Livingston N Heywood JT Correlates of health-related quality of life in patients with heart failure Heart Lung 2002 31 2 85 93 11910383 10.1067/mhl.2002.122839

[B27] Beery TA Baas LS Fowler C Allen G Spirituality in persons with heartfailure J Holist Nurs 2002 20 1 5 25 11898688 10.1177/089801010202000102

[B28] Erah PO Arute JE Adherence of HIV/AIDS patients to antiretroviral therapy in a tertiary health facility in Benin City Afr J Pharm Pharmacol 2008 2 7 145 152

[B29] Jesse DE Reed RG Effects of spirituality and psychosocial well-being on health risk behaviors in Appalachian pregnant women J Obstet, Gynecolo, and Neonatal Nurs 2004 33 739 747 10.1177/0884217504270669 15561662

[B30] Fetzer Institute, National Institute on Aging Working Group Multidimensional measurement of religiousness/ spirituality for use in health research 2003 Kalamazoo: Fetzer Institute

[B31] Ross S Walker A MacLeod MJ Patient compliance in hypertension: role of illness perceptions and treatment beliefs J Hum Hypertens 2004 18 607 613 15029218 10.1038/sj.jhh.1001721

[B32] Morisky DE Ang A Krousel-Wood M Ward HJ Predictive validity of a medication adherence measure in an outpatient setting J Clin Hypertens 2008 10 5 348 354 10.1111/j.1751-7176.2008.07572.x PMC2562622 18453793

[B33] Simoni JM Frick PA Huang B A longitudinal evaluation of a social support model of medication adherence among HIV-positive men and women on antiretroviral therapy Health Psychol 2006 25 1 74 81 16448300 10.1037/0278-6133.25.1.74 PMC5096446

[B34] Raghavan R Ferlic-Stark L Clarke C Rungta M Goodgame R The role of patient religiosity in the evaluation and treatment outcomes for chronic HCV Infection J Rel Health 2013 52 79 90 10.1007/s10943-011-9455-y 21246281

[B35] Wanyama J Castelnuovo B Wandera B Mwebaze P Kambugu A Bandsberg DR Belief in divine healing can be a barrier to antiretroviral therapy adherence in Uganda AIDS 2007 21 11 1486 1487 17589198 10.1097/QAD.0b013e32823ecf7f

[B36] Johnstone B Franklin KL Yoon DP Burris J Shigaki C Relationships among religiousness, spirituality, and health for individuals with stroke J Clin Psychol Med Settings 2008 15 308 313 19104988 10.1007/s10880-008-9128-5

[B37] de-Graft Aikins A Anum A Agyemang C Addo J Ogedegbe O Lay representations of chronic diseases in Ghana: implications for primary prevention Ghana Med J 2012 46 2 59 68 23661819 PMC3645147

[B38] Read U “I want the one that will heal me completely so it won’t come back again”: the limits of antipsychotic medication in rural Ghana Transcult Psychiatry 2012 49 3–4 438 460 22722982 10.1177/1363461512447070

